# A Holistic View of Dietary Carbohydrate Utilization in Lobster: Digestion, Postprandial Nutrient Flux, and Metabolism

**DOI:** 10.1371/journal.pone.0108875

**Published:** 2014-09-30

**Authors:** Leandro Rodríguez-Viera, Erick Perera, Antonio Casuso, Rolando Perdomo-Morales, Odilia Gutierrez, Idania Scull, Olimpia Carrillo, Juan A. Martos-Sitcha, Tsai García-Galano, Juan Miguel Mancera

**Affiliations:** 1 Center for Marine Research, University of Havana, Havana, Cuba; 2 Instituto de Ciencias Marinas de Andalucía, ICMAN-CSIC, Puerto Real, Cadiz, Spain; 3 Biochemistry Department, Center for Pharmaceuticals Research and Development, Havana, Cuba; 4 Institute for Animal Science, Mayabeque, Cuba; 5 Faculty of Biology, University of Havana, Havana, Cuba; 6 Department of Biology, Faculty of Marine and Environmental Sciences, Campus de Excelencia Internacional del Mar (CEI-MAR), University of Cadiz, Puerto Real, Cadiz, Spain; The Evergreen State College, United States of America

## Abstract

Crustaceans exhibit a remarkable variation in their feeding habits and food type, but most knowledge on carbohydrate digestion and utilization in this group has come from research on few species. The aim of this study was to make an integrative analysis of dietary carbohydrate utilization in the spiny lobster *Panulirus argus*. We used complementary methodologies such as different assessments of digestibility, activity measurements of digestive and metabolic enzymes, and post-feeding flux of nutrients and metabolites. Several carbohydrates were well digested by the lobster, but maize starch was less digestible than all other starches studied, and its inclusion in diet affected protein digestibility. Most intense hydrolysis of carbohydrates in the gastric chamber of lobster occurred between 2–6 h after ingestion and afterwards free glucose increased in hemolymph. The inclusion of wheat in diet produced a slow clearance of glucose from the gastric fluid and a gradual increase in hemolymph glucose. More intense hydrolysis of protein in the gastric chamber occurred 6–12 h after ingestion and then amino acids tended to increase in hemolymph. Triglyceride concentration in hemolymph rose earlier in wheat-fed lobsters than in lobsters fed other carbohydrates, but it decreased the most 24 h later. Analyses of metabolite levels and activities of different metabolic enzymes revealed that intermolt lobsters had a low capacity to store and use glycogen, although it was slightly higher in wheat-fed lobsters. Lobsters fed maize and rice diets increased amino acid catabolism, while wheat-fed lobsters exhibited higher utilization of fatty acids. Multivariate analysis confirmed that the type of carbohydrate ingested had a profound effect on overall metabolism. Although we found no evidence of a protein-sparing effect of dietary carbohydrate, differences in the kinetics of their digestion and absorption impacted lobster metabolism determining the fate of other nutrients.

## Introduction

Decapod crustaceans live in virtually all marine and freshwater habitats on Earth (also a few species are largely terrestrial), and exhibit a remarkable variation in their feeding behaviors, from filter feeding, scavenging, grazing to hunting, and in the composition of their diet. However, most knowledge about crustacean digestion has come from studies on a few economically relevant decapods due to the importance of optimized formulated feeds for aquaculture success [1]–[3]. Vegetal-derived flours and starches (native or processed) have been historically used in aquafeeds due to their reasonable price and good results for most species. Starches are, in general, well digested by decapod crustaceans [1], including spiny lobsters [2]–[6]. However, starch digestibility in shrimp varies from 60% to 96% [1] and in spiny lobsters from 59% (maize) to 91% (wheat) [3]. Those variations depend not only on several features of the starch itself [7]–[10], but also on the level of inclusion in diet [11], the throughput rate of the digesta [11], and the activity of digestive carbohydrases [12]–[22].

While digestibility values are indicative of overall digestion, the time-course emergence of digestion products may vary among crustacean taxa, and within the same species for different carbohydrates. High carbohydrate hydrolysis and glucose absorption are widely recognized in crustaceans [1], [23]–[26], but differences occur among different crustacean taxa concerning the location of carbohydrate digestion. The highest amylase activity in shrimp is found within the digestive gland [17], while amylase activity is largely found in the gastric juice of spiny lobsters (*P. argus* [21], *J. edwardsii* [12]), and other lobster species such as *Homarus gammarus* [18]. The effects of the time-course appearance of carbohydrate digestion products on the energy metabolism remain unknown.

The utilization of glycogen and free glucose has been studied in crustaceans under different stressful conditions [6], [26]–[29], but the role of carbohydrates during regular feeding is less understood. This issue is particularly interesting in spiny lobsters given the occurrence of high and prolonged hyperglycemia after a meal, which in some species may last 30 h [2]. In shrimp, carbohydrates can spare to a certain extent the dietary proteins in spite of prolonged hyperglycemia [30] by changing the metabolic substrate from protein to a mixture of protein, lipids and carbohydrates [23]. However, there is no direct evidence of a significant use of carbohydrates for energy in spiny lobsters. Spiny lobsters have a marked protein-based energy metabolism [31], [32], and have little glycogen stored in their digestive gland [5], [6], [33]. A slight protein-sparing effect could be demonstrated in *P. argus* only for lipids under certain dietary conditions [31], [32]. This information for lobster, however, was gathered from analysis of the oxygen consumption: ammonia excretion atomic ratio (O:N), without direct evidence at the level of intermediary metabolism. Several studies have evaluated different metabolic, mostly glycolytic, enzymes in economically relevant crustaceans such as penaeid shrimp in relation to carbohydrate level in diet [24], [34], [35], starvation [29], [36], molt cycle [37], and osmotic stress [24], [35], but there are no previous reports on the activities of these enzymes in spiny lobster in relation to feeding.

In addition, there is poor information on the post-prandial flux of nutrients (i.e., amino acids, lipids) other than glucose [2], [4] after feeding in spiny lobsters and other crustaceans, which may also affect the use of dietary carbohydrate. Even for penaeid shrimps, the nutritionally most studied crustaceans during the past four decades, to our knowledge, there is only one report on the post-prandial changes of amino acids in hemolymph [38]. Although several studies are available on spiny lobster nutrition [4], [5], [39]–[47], the role of dietary carbohydrate on energy metabolism and their interactions with other nutrients remains largely unknown in comparison to more thoroughly studied crustaceans such as penaeid shrimp.

The aim of this study was to evaluate the effects of the extent and the time-course of carbohydrate digestion on the metabolism of the spiny lobster *P. argus*. We used complementary methodologies such as i) *in vitro* and *in vivo* assessments of digestibility, ii) measurements of amylase activity in the digestive tract, iii) post-feeding flux of nutrients and metabolites, and iv) determination of activity of key metabolic enzymes, in a step by step approximation to dietary carbohydrate utilization in lobster. While, as widely accepted, the use of carbohydrates by crustaceans depends on their overall digestibility, we showed that differences in carbohydrate digestion kinetics have a profound impact on lobster metabolism and on the utilization of other nutrients. Results presented may assist in selecting physiologically appropriated carbohydrate sources for *P. argus*, and constitute baseline data for further optimization of formulated feeds in this species.

## Materials and Methods

### Preparation of digestive gland extracts

Spiny lobsters were collected in the Gulf of Batabanó, Cuba, by SCUBA diving. The collection area was: 21°39.0431′N–83°09.8436′W; 21°41.0015′N–83°092463′W; 21°40.1016′N–83°11.0297′W, and it was performed under permission of the Fisheries Regulator Department from the Ministry of the Fishing Industry of Cuba. This study did not involve endangered or protected species. Lobsters were anesthetized by immersing them into ice-cold water before digestive gland extraction. Samples were immediately frozen in liquid nitrogen and stored at −80°C. Tissue was homogenized with chilled Milli-Q water (90 mg/500 µL) using a glass piston homogenizer, and the homogenate centrifuged at 10 000×g for 30 min at 4°C. The resultant upper lipid layers were discarded and the remaining supernatants were stored at −80°C until used for *in vitro* digestion assays, after determination of its amylase activity.

### Amylase activity

Amylase activities were determined using an HELFA Amilase Assay Kit (Quimefa Biologic Products Inc. Havana, Cuba) with CNPG_3_ (2-Chloro-4-nitrophenyl-α-D-maltotrioside) as the substrate, following the manufacturer's instructions. One unit of amylase activity was defined as the amount of enzyme that produces the release of 1 µmol nitrophenol per minute. Units of amylase activity were expressed per volume, weight of tissue, or soluble protein as needed.

### Protein concentration

Soluble protein concentrations were quantified according to [48] using bovine serum albumin as the standard.

### *In vitro* digestion in Eppendorf tubes

Carbohydrate digestibility was assessed *in vitro* in term of glucose released after incubation with digestive gland extracts. Thirteen different carbohydrates sources (300 mg) (Table 1) were dissolved each in 5 mL of Milli-Q water to achieve a concentration of 6% (w/v) and used as substrate solutions as described before [2]. The amount of digestive gland extracts added for each digestion assay (N = 30, per carbohydrate source) were adjusted in term of amylase activity in order to assure similar extract composition against the substrates. *In vitro* assays were performed using a method previously described [1] and modified [2] as follows: 250 µL of solutions or suspensions of the carbohydrate substrates (Table 1) and digestive gland extracts (0.2 U of final amylase activity in the mixture) were mixed and diluted up to 1 mL with 100 mM citrate-phosphate buffer (pH 5.0) in 2 mL Eppendorf tubes. Tubes were shaken for 60 min at room temperature (26°C) [the rate of carbohydrate hydrolysis was linear up to two hours of incubation under this assay conditions, data not shown]. Then, 20 µL of samples were taken and stored at −20°C for glucose determination.

**Table 1:** Carbohydrate sources used for *in vitro* digestion.

Carbohydrates	Source
Agar	Sigma-Aldrich (Fluka), cat. No. 05039
Agarose	SERVA Electrophoresis GmbH, cat. No. 11403
Alginate	Sigma-Aldrich, cat. No. W201502
Carboxymethyl cellulose	Sigma-Aldrich cat. No. C5678
Glycogen	Sigma-Aldrich, cat. No. G8751
Maize starch*	Indias, Baldinelli G.R., Argentina
Potato starch*	AppliChem-Panreac, cat No. A2223
Rice starch	BDH, Merck Chemicals Ltd., cat. No. 30263
Maize flour	Yellow, Fine, Iberia Foods Corp., Brooklyn, NY
Rice flour	Made at the laboratory by finely milling white rice
Wheat flour	Commercially available regular foodstuff

^*^Marked carbohydrates were also assayed after gelatinisation at 80°C for 20 min (100 g L^−1^ deionizer water) and dried at 50°C for 48 h [2].

Glucose released was determined using a HELFA RapiGluco-Test glucose oxidase Kit (Quimefa Biological Products Inc., Havana, Cuba) following the manufacturer's instructions. The assays were performed in duplicate. Blank assays without addition of enzyme extracts or the substrates were carried out for each carbohydrates source to estimate the amount of free glucose present in the extracts and the substrates. The hydrolysis rate (HR) [2] of each substrate was calculated as: HR (nmol glucose min^−1^)  =  ([glucose]_F_ − [glucose]_0_)/t, where [glucose]_F_ is the final glucose concentration after incubation with enzyme extracts, [glucose]_0_ is the glucose concentration present collectively in the substrate and enzyme extracts, and t is the incubation time in minutes. As glucose is not the only product of carbohydrate hydrolysis, reducing sugars were also determined by the Somogy-Nelson method [49] using maltose as the standard, to express HRs also as mg maltose equivalent released per minute.

### *In vivo* digestibility by the inert marker chromic oxide

Apparent *in vivo* digestibility was assessed using 1% of the inert marker chromic oxide in formulated diets (Table 2). Three experimental isoenergetic diets were formulated to have 45% protein, 10% lipids, and 35% of three different carbohydrates (rice starch, wheat flour, maize starch) of suspected different digestibilities according to previous *in vitro* results. All feedstuffs were obtained from commercial suppliers, except fish and squid meals that were made at the laboratory as described before [50]. Pellets were made as described previously [51], but extruded twice for a better homogenization of the marker.

**Table 2:** Formulation (%) and proximate composition of the experimental diets.

Ingredients	Wheat diet	Maize diet	Rice diet
Fish meal^a^	31	35	35
Squid meal^a^	13.2	17	17
Gelatin^b^	5	5	5
Wheat flour^c^	40.9	-	-
Maize starch^d^	-	30	-
Rice starch^e^	-	-	30
Fish oil^f^	1.9	1.9	1.9
Lecithin^g^	2	2	2
Cholesterol^h^	1	1	1
Vit & Min premix^i^	1	1	1
Chromic oxide^j^	1	1	1
Phosphate/carbonate^k^	2	2	2
Attractants^l^	1	1	1
Talc^c^	-	3.1	3.10
Total	100	100	100
Proximate composition^m^			
Crude protein	46.5	46.6	45.9
Crude lipid	10.5	9.7	9.7
Carbohydrate	36.4	35.8	35.7
Ash	7.4	11.3	7.4

Pellets contained 10–12% of water.

a Prepared at the laboratory as detailed before [50]. Jack mackerel meal: 79.1% proteins, 16.8% lipids, 5.5% moisture; Squid meal: 76.6% proteins, 10.8% lipids, 8.7% moisture.

b Sigma-Aldrich (G2500).

c Commercially available regular feedstuff.

d Indias, G.R. Baldinelli, Argentina.

e BDH (30263), Merck Chemicals Ltd.

f Fisheries Research Center Laboratory, Havana, Cuba.

g Calbiochem (429415), Merck Chemicals Ltd.

h Sigma-Aldrich (C8667).

i Premix from DIBAQ-Aquaculture, Segovia, Spain, containing (per kg of feed): vitamin A 15,000 IU, vitamin D3 3000 IU, vitamin E 180 mg, vitamin K 15 mg, vitamin B1 37.5 mg, vitamin B2 37.5 mg, vitamin B6 24.75 mg, vitamin B12 0.045 mg, vitamin H 1.14 mg, D-pantothenic acid 120 mg, nicotinic acid 225 mg, vitamin C 300 mg, folic acid 11.24 mg, Inositol 112.5 mg, zinc 75 mg, selenium 0.3 mg, magnesium 86.25 mg, copper 2.25 mg, manganese 22.5 mg, iodine 7.5 mg, iron 3 mg, cobalt 0.3 mg.

J Cr_2_O_3_ (Sigma-Aldrich, 393703).

k Dicalcium phosphate/Calcium carbonate (1∶2), Santa Cruz Fish Feed Factory, Camagüey, Cuba.

l Taurine (Sigma-Aldrich, T0625) 500 mg/Kg diet, Glycine (Sigma-Aldrich, G8898) 500 mg/Kg diet.

m Measured as described in Materials and Method section. Crude lipid calculated from proximate composition of ingredients.

Spiny lobsters (90–150 g) were collected as described above and transported alive to the Center for Marine Research of the University of Havana, Cuba. Only apparently healthy late-intermolt (C4) specimens (determined according to Lyle and MacDonald [52]) were used. The feeding trial was conducted for 30 days in an indoor facility equipped with recirculated sea water, sand and biological filtration, constant aeration, and photoperiod cycle of 12 h light: 12 h dark. Water quality was monitored in the morning twice a week for temperature (∼26°C), pH (∼8.0), salinity (36‰), dissolved oxygen (∼6.0 mg/L), and ammonia-N (∼0.07 mg/L). Each of the three experimental diets was sorted at random to six lobsters (N = 6, per diet), housed individually in 60-L tanks.

Lobsters were acclimated for one week to the experimental diets by gradually reducing fish flesh until they consumed exclusively the pellets. The amount of diet given was progressively adjusted to 2% of body weight per day (BW day^−1^) according to the appetite of the lobsters by checking the bottom of the tanks for excess feed remaining one hour after feeding. This feeding time is enough for lobsters fed close to satiation [53]. Following one week of acclimation to the experimental diets, each lobster was fitted with a fecal collection devise [54] modified as in [3]. Lobsters were fed one ration a day in the morning. Feces were collected once daily, one hour after feeding to reduce the impact of handle stress on feed intake [54]. Feces was carefully removed from the tubes and stored at −20°C. Successive fecal samples collected from the same lobster were pooled together until the total collection of around 100 g of wet feces per lobster [equivalent to about 2 g of dry matter (DM)]. Diets were rotated each week trough the three group of lobsters to avoid adaptation, with two days of fasting in-between. Any collection that was contaminated with sea water was discarded. Composition analyses of diets and feces were performed according to [55]. Briefly, dry matter was analyzed by weight change following drying at 105°C to a constant weight, ash was determined by weight change following furnace incineration at 550°C for 5 h, total protein was determined by the Kjeldahl method, total carbohydrates were measured by the acid hydrolysis method, and chromic oxide was determined by the perchloric acid digestion method [55].

Apparent digestibility (AD) was calculated as in [1]: AD (%)  = 100× [1– (c_i_/c_f_) × (n_f_/n_i_)], where c_i_ and c_f_ are the concentrations (dry matter basis) of chromic oxide in the ingested diet and feces respectively, and n_i_ and n_f_ are the concentrations (dry matter basis) of the nutrient in the ingested diet and feces, respectively.

### Serial collection of gastric juice and hemolymph

Another three groups of five lobsters were adapted to experimental diets as above and fed for one week. Later, they were fasted for 2 days and were provided with a ration of the experimental diets for gastric juice and hemolymph collection. Gastric fluid samples were obtained through the oral cavity using disposable insulin syringes with a plastic cannula over the sharp end of the needle as described before [51]. Gastric juice was not sampled prior to feeding as this was presumed to affect feed intake due to stress. Samples (∼100 µL) of gastric juice were taken from the same lobster at 2, 6, 12, 24 and 30 h after ingestion, centrifuged at 10,000×g for 10 min, frozen in liquid nitrogen and then stored at −80°C. Lobsters were handled with care and samples were rapidly taken (less than 1 min) to avoid excessive stress. We previously demonstrated that this serial sampling of gastric juice produces no variation in hemolymph glucose concentration as a result of manipulation [51]. Hemolymph was neither sampled prior to feeding as this is known to affect feed intake in other spiny lobster species [2]. Hemolymph sampling began 2 h after feeding, with additional samples at 6, 12, 24, and 30 h from each lobster. Previous studies in spiny lobsters revealed that the effect of serial sampling of hemolymph on hemolymph glucose concentration is negligible [56]. Hemolymph samples (500 µL) were taken from the sinus of the 4^th^ walking legs [57] in 1 mL pyrogen free disposable syringes containing 500 µL of precooled anticoagulant solution (400 mM NaCl, 10 mM KCl, 10 mM Hepes, 20 mM EDTA, pH 7.3) [58]. An additional group of five lobsters were left unfed and sampled as above, corroborating that no variation in the variables studied occurs due to manipulation or daily rhythm.

### Time-course of nutrients and metabolites in gastric juice and hemolymph

Increasing soluble protein in gastric juice was measured as indicator of solubilization of dietary protein plus enzyme secretion into the foregut. Gastric juice glucose and free amino acids were measured as indicators of rates of carbohydrate and protein hydrolysis in the foregut. Gastric juice triglycerides were measured as indicator of lipid solubilization/emulsification from feeds. The glycemic prandial response was analyzed as indicator of digestibility and assimilation of dietary carbohydrates in lobsters [2], [56], and the same rational was applied for free amino acids and triglycerides in hemolymph. Soluble protein and glucose concentrations were quantified as described above. Free amino acid levels were assessed colorimetrically using the nynhidrin method [59], [60] using L-alanine as the standard. Triglyceride and lactate concentrations were measured using the commercial kits TAG (Spinreact, Girona, Spain) and Lactate (Spinreact, Girona, Spain), respectively.

### Metabolites in digestive gland and muscle

At the end of the 30 h time-course sampling of gastric juice and hemolymph, the three groups of lobsters were fed during one month with the same experimental diets without disturbance, left unfed for 48 h, and then fed again with the respective diets. They were sacrificed 24 h later in ice-cold water to obtain digestive gland and muscle samples. Five additional lobsters that were fed during one month with fish flesh were also left unfed for 48 h, sampled as above, and referred as fresh fish treatment. Samples were immediately frozen in liquid nitrogen and then lyophilized for metabolite and metabolic enzyme measurements (see below). For metabolites, powder of digestive gland and muscle were weighed and homogenized in water (∼20 mg/mL), centrifuged (30 min at 10,000×g, 4°C), and the supernatant was used to assess tissue metabolites. Prior to centrifugation, an aliquot was removed and frozen at −80°C for triglyceride determination. Protein, glucose, free amino acid, triglyceride, and lactate were measured as above. Glycogen concentration was assessed as described before [61].

### Metabolic enzymes in digestive gland and muscle

The activities of metabolic enzymes from different pathways were quantified in two key tissues in crustacean metabolism, digestive gland and muscle. The digestive gland of crustaceans is the main site for the synthesis of digestive enzymes, digestion, absorption, metabolism and storage of nutrients, as well as for their mobilization [62], thus being metabolically very active. Gland respiration is known to increase in 56% during the first 6 h after feeding in some crustaceans (e.g. shrimp [63]). Muscle, on the other hand, sustains locomotor activity and growth. Lyophilized samples of digestive gland and muscle were homogenized in 10 volumes of ice-cold buffer containing 50 mM imidazole hydrochloride (pH 7.5), 1 mM 2-mercaptoethanol 50 mM sodium fluoride, 4 mM EDTA, 250 mM sucrose, and 0.5 mM PMSF. Homogenates were centrifuged for 30 min at 10,000×g and supernatants used for assays. Enzymes examined were: hexokinase (HK, EC 2.7.1.11), glycerol-3-phosphate dehydrogenase (G3PDH, EC 1.1.1.8), pyruvate kinase (PK, EC 2.7.1.40), L-lactate dehydrogenase (LDH, EC 1.1.1.27), fructose 1,6-biphosphatase (FBPase, EC 3.1.3.11), glycogen phosphorylase (GPase, EC 2.4.1.1), glucose-6-phosphate dehydrogenase (G6PDH, EC 1.1.1.49), aspartate transaminase (AST, EC 2.6.1.1), alanine transaminase (ALT, EC 2.6.1.2), glutamate dehydrogenase (GDH, EC 1.4.1.2), and 3-hydroxyacyl-CoA dehydrogenase (HOAD, EC 1.1.1.35). Amount of sample in each assay was set in preliminary tests to ensure initial velocities for all enzymes studied. Conditions for enzyme assays [e.g. buffer composition, cofactors, additional enzymes for coupled enzyme assays, and electron donor (NADH) or acceptors (NADP, NAD)] were as described elsewhere [65], [64]. Substrates were: 5 mM D-glucose for HK, 0.2 mM dihydroxyacetone phosphate for G3PDH, 5 mM D-glucose PK, 6.25 mM lactic acid for LDH, 0.1 mM fructose-1,6-bisphosphate for FBPase, 5 mg/mL glycogen for GPase, 1 mM glucose-6-phosphate for G6PDH, 10 mM L-aspartate for AST, 7.5mM L-alanine for ALT, 1.40 mM α-ketoglutarate for GDH, and 0.1 mM acetoacetyl-CoA for HOAD. Reactions without substrates were also performed as controls. Reaction rates of the enzymes HK, LDH, FBPase, GPase, and G6PDH were determined in duplicate by the increase in absorbance at 340 nm and 37°C as a result of the formation of NADPH. Reaction rates of the enzymes G3PDH, PK, AST, ALT, GDH, HOAD were determined in duplicate by the decrease in absorbance at 340 nm and 37°C as a result of the disappearance of NADH.

All assays were performed using a Bio-Tek PowerWave 340 Microplate spectrophotometer using KCjunior Data Analysis Software (Bio-Tek Instruments, Winooski, VT, USA). One unit of enzyme activity (U) was defined as the amount of enzyme needed to transform 1 µmol of substrate or produce 1 µmol of product per minute. Enzyme activity was expressed as specific activity as U/mg protein.

### Statistical analyses

Only results from late-intermolt (C4) lobsters were analyzed as molt stage has been found to affect digestive enzyme activities [22] and feeding activity [66] of *P. argus*. All data were checked for normality and homogeneity of variance using Kolmogorov-Smirnov and Levene's tests, respectively, with P≤0.05. Logarithmic transformations of data were made when necessary to fulfill the assumptions of ANOVA. *In vitro* rates of hydrolysis (N = 30 lobsters, per carbohydrate) and *in vivo* apparent digestibilities (N = 6 lobsters, per dietary treatment) were analyzed by one-way ANOVA (P≤0.05), being the carbohydrate substrates and the experimental diets the sources of variation, respectively. Metabolic enzyme activities and metabolites in digestive gland, hemolymph, and muscle 24 h after ingestion were also analyzed by one-way ANOVA (P≤0.05). All time-course data were subjected to two-way ANOVA (P≤0.05), with diet and time being the two sources of variation. In all cases, the Tukey's test (P≤0.05) was used to determine differences among means.

Those variables that significantly varied among diets 24 h after feeding according to univariate analysis detailed above were assumed to be of most predictive value, thus selected for multivariate analysis. We performed a forward stepwise discriminant analysis for two sets of variables, metabolic enzyme activities and metabolites, to understand the combination of variables that can best explain the response of lobsters to diet (carbohydrate) ingested. For the analysis, it was considered F to enter as 0.01, F to remove at 0.0, and minimum tolerance of 0.01. After significant functions development, the relative importance of the original variables in separating the diet ingested by lobster was gauged by standardised values. The software package Statistica 7.0 (StatSoft Inc., Tulsa, OK, USA) was used for all tests performed and figures were generated by GraphPad Prism 5.00 (GraphPad Software, Inc., San Diego, California, US).

## Results

### *In vitro* hydrolysis rate of carbohydrate

The liberation of glucose, except for the rice starch, was a suitable measure of carbohydrate digestion as glucose and reducing sugars released, measured by the Somogy-Nelson method, were significantly correlated [mg glucose  = .64+9703 (mg maltose equivalents), F = 63.56, P = 0.004, R^2^ = 0.95]. The observed values of glucose released from rice starch were 5 times higher than the ones expected from the correlation between glucose and reducing sugars.

**Figure 1:**
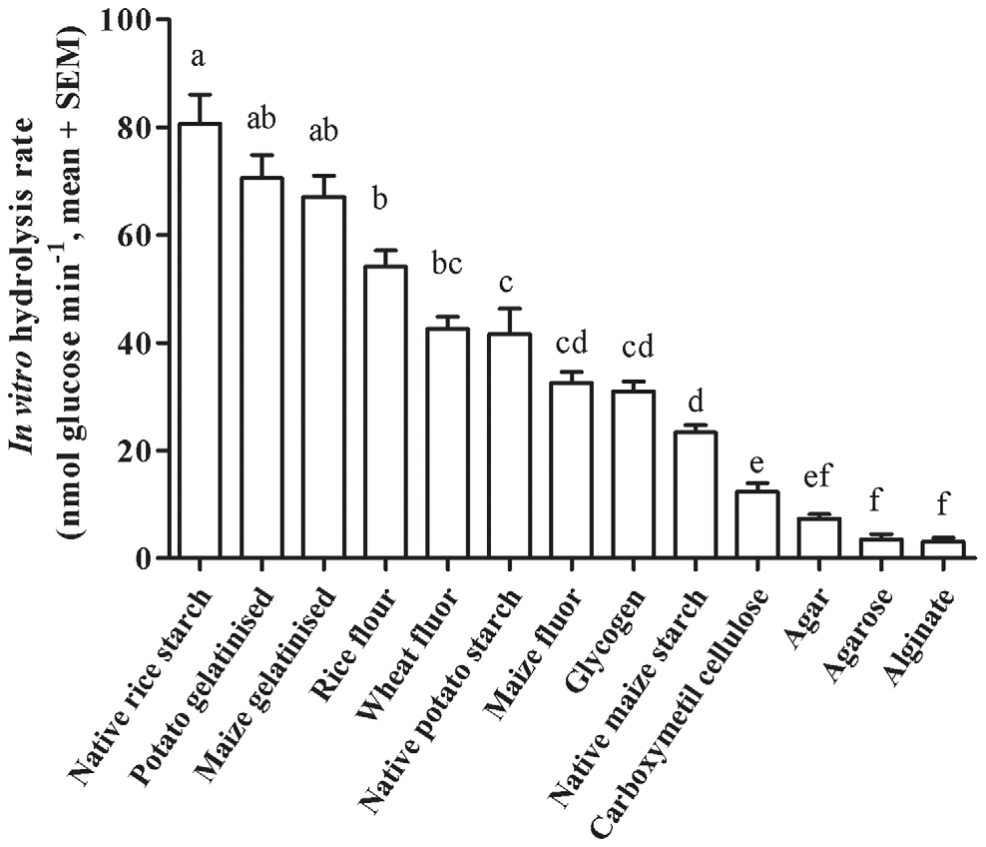
*In vitro* hydrolysis rates of different carbohydrate substrates by digestive gland extracts of the spiny lobsters *Panulirus argus*. Each value is the mean ± SEM (N = 30 lobsters per diet) Significant differences are marked by different superscript letters (one-way ANOVA, Tukey test, P≤0.05).

*In vitro* digestion of all the carbohydrates sources tested by digestive gland extracts of the spiny lobster *P. argus* resulted in the liberation of glucose (Fig. 1). The hydrolysis rates (HRs) of the carbohydrate substrates tested were significantly different (one-way ANOVA, F = 68.34, P≤0.001) (Fig. 1). Native rice starch displayed the highest HR (nmol glucose min^−1^, mean ± SEM) of all the carbohydrate substrates tested (80.60±5.46) (Fig. 1). Other carbohydrates were also digested at a high rate such as gelatinised potato starch (70.5±4.29) and gelatinised maize starch (67.0±3.94). Intermediate HRs were obtained for rice flour (54.1±2.98), wheat flour (42.6±2.29), potato starch (41.6±4.79), maize flour (32.5±2.06), glycogen (30.9±1.87), and maize starch (23.2±1.40) (Fig. 1). The lowest HRs were found for carboxymethyl cellulose (12.3±1.61), alginate (3.3±0.70), agarose (3.2±0.98), and agar (7.4±0.83) (Fig. 1).

### *In vivo* apparent digestibility

Responses *in vivo* may diverge from those predicted *in vitro*, thus we assessed *in vivo* digestibility in this study to ensure that selected carbohydrates (rice starch, wheat flour and maize starch) for studying post-absorptive process in lobster indeed differ in digestibility as predicted *in vitro*. There were no statistically significant differences in apparent dry matter digestibility among experimental diets (F = 0.61, P>0.05) (Table 3). Conversely, carbohydrate (F = 7.92, P≤0.05) and protein (F = 41.99, P≤0.001) *in vivo* apparent digestibilities were significantly lower for the maize diet than for the other two diets (Table 3).

**Table 3:** Dry matter, carbohydrate, and crude protein apparent digestibility of formulated diets containing 45% protein, 9% lipids, and 30% of different carbohydrate sources (wheat flour, rice starch, maize starch) fed to *Panulirus argus*.

Diet	Dry matter (%)	Carbohydrate (%)	Crude protein (%)
Wheat flour	46.4±7.23	90.7±4.06^a^	83.0±4.12^a^
Rice starch	47.8±2.78	81.4±3.96^ab^	70.6±4.48^a^
Maize starch	38.7±8.98	60.1±1.06^b^	37.6±1.68^b^

Values are means ± SEM (N = 6). Significant differences within the same column are indicated by different letters (one-way ANOVA, Tukey test, P≤0.05).

### Soluble proteins and amylase activity in gastric juice

**Figure 2:**
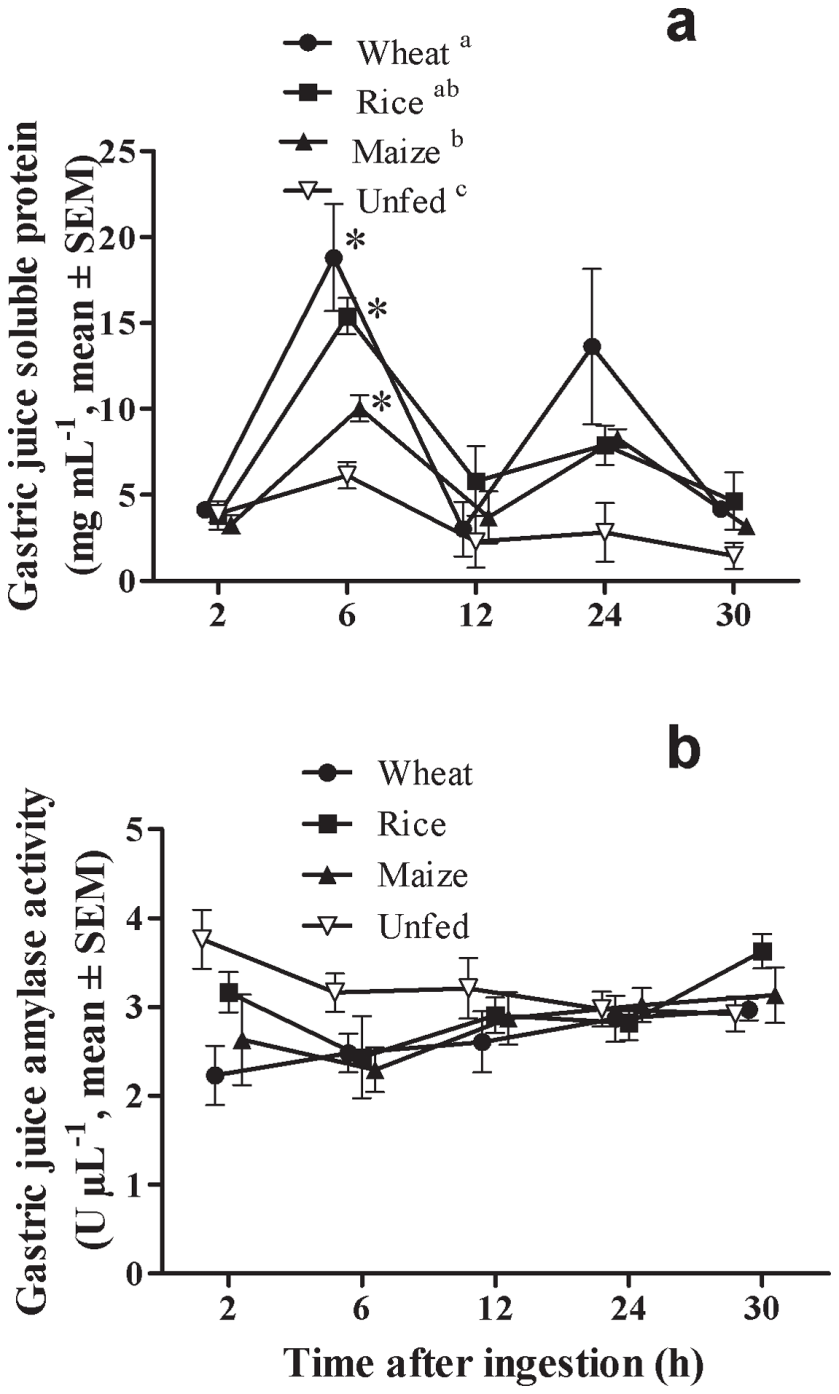
Changes in concentrations of soluble protein (a) and α-amylase activity (b) in the gastric juice of *Panulirus argus* after feeding diets with different carbohydrate sources. Diets were named according to the carbohydrate source they contained (wheat flour, rice starch, maize starch). Each value is the mean ± SEM (N = 5 lobsters per diet). For each dietary treatment, the time at which the first statistically difference was found with respect to 2 h is marked by an asterisk. Differences among diets throughout the 30-h experiments are marked by different superscript letters in the legend (Two-way ANOVA, Tukey test, P≤0.05).

Soluble proteins in the gastric juice significantly varied through time (Two-way ANOVA, F = 28.04, P≤0.001), and among diets (Two-way ANOVA, F = 4.40, P≤0.05), whereas no significant interaction was found between these factors (Two-way ANOVA, F = 2.13, P>0.05). Two peaks of soluble proteins were found in the gastric juice at 6 and 24 h after ingestion all the experimental diets (Fig. 2a). Six hours after ingestion, there were differences among diets (One-way ANOVA, F = 4.75, P≤0.05) in soluble protein concentration of the gastric juice, being significantly higher for wheat than for maize diet (Tuckey's test, P≤0.05) (Fig. 2a). Twenty-four hours after the last meal there were no differences among diets in soluble protein concentration in the gastric juice (One-way ANOVA, F = 2.06, P>0.05), despite lobsters fed with the wheat diet tended to have more soluble proteins in their gastric juice (Fig. 2a). There were no significant differences in amylase activity per volume of gastric juice trough time (Two-way ANOVA, F = 2.49, P>0.05) and among diets (Two-way ANOVA, F = 2.23, P>0.05), neither interaction among these factors (Two-way ANOVA, F = 0.59, P>0.05) (Fig. 2b).

### Time-course of glucose in gastric juice and hemolymph after feeding

**Figure 3:**
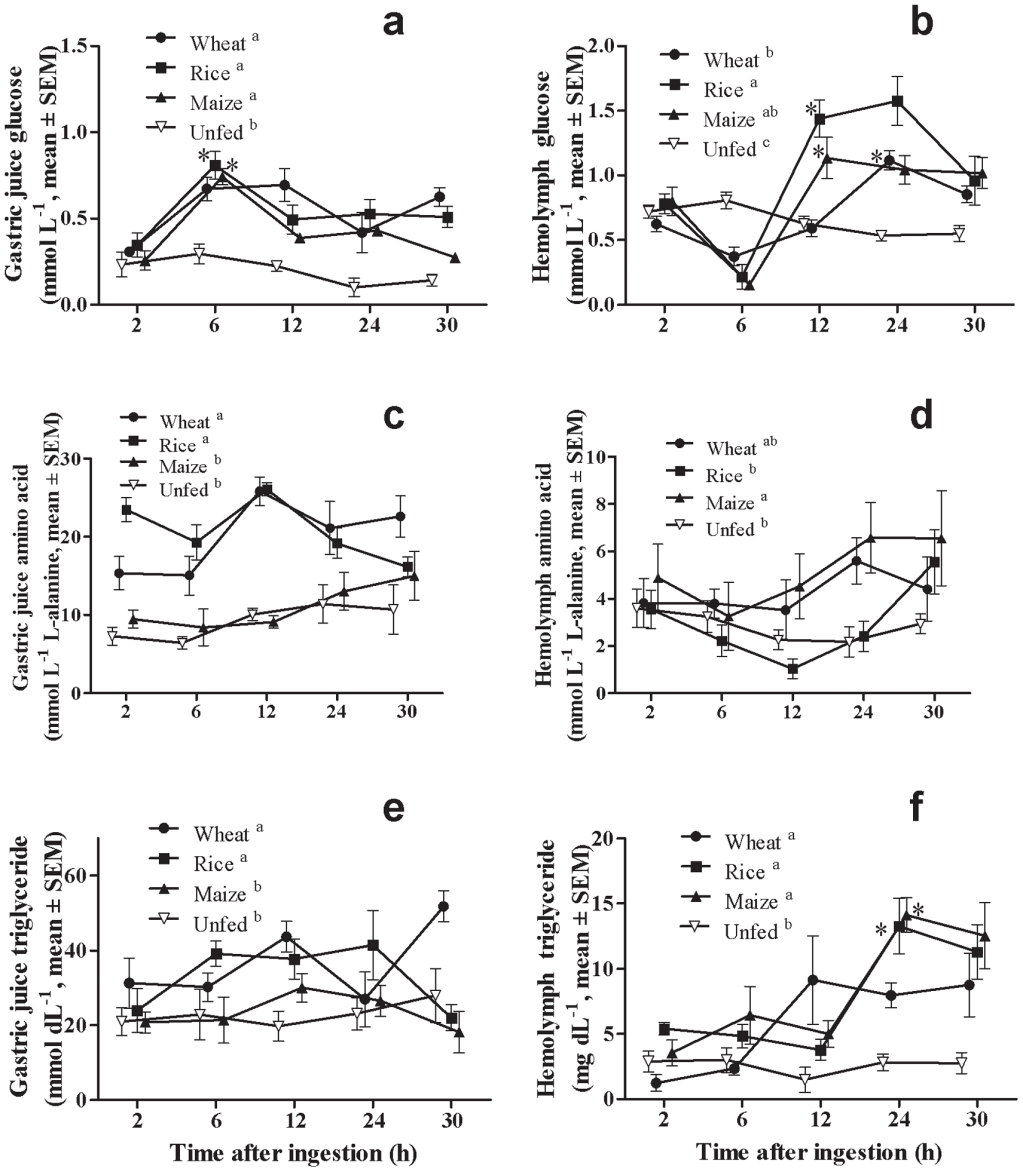
Changes in concentrations of glucose in gastric juice (a) and hemolymph (b), amino acid in gastric juice (c) and hemolymph (d), and triglyceride in gastric juice (e) and hemolymph (f) of *Panulirus argus* after feeding diets with different carbohydrate sources. Diets were named according to the carbohydrate source they contained (wheat flour, rice starch, maize starch). Each value is the mean ± SEM (N  = 5 lobsters per diet). For each dietary treatment, the time at which the first statistically difference was found with respect to 2 h is marked by an asterisk. Differences among diets throughout the 30-h experiments are marked by different superscript letters in the legend (Two-way ANOVA, Tukey test, P≤0.05).

Free glucose in the gastric juice varied through time of digestion with maximal values 6 h after ingestion (Two-way ANOVA, F = 10.89, P≤0.001), and differed between fed and unfed lobsters (Two-way ANOVA, F = 7.34, P≤0.01) (Fig. 3a). No interaction between diet and time was found (Two-way ANOVA, F = 1.88, P>0.05). Lobsters that ingested the wheat diet tended to have a higher free glucose concentration in the gastric juice 12 h after meal respect to those fed the other diets suggesting a delay in absorption, although no statistical differences were found (Fig. 3a). On the other hand, there were differences among diets (Two-way ANOVA, F = 5.75, P≤0.05) and through time (Two-way ANOVA, F = 28.09, P≤0.001) in hemolymph free glucose levels, resulting the interaction between both factors also significant (Two-way ANOVA, F = 3.47, P≤0.05) (Fig. 3b). Glucose concentration in hemolymph increased with maximal values attained at 12 h (for rice and maize diets) or 24 h (for wheat diet) after ingestion (Fig 3b). Concentration of glucose in hemolymph 12 h after ingestion was significantly lower (One-way ANOVA, F = 23.91, P≤0.05; Tukey's test, P≤0.05) in wheat fed lobsters than in lobsters ingesting the other diets (Fig. 3b).

### Time-course of free amino acid in gastric juice and hemolymph after feeding

Free amino acid concentration in the gastric juice did not varied through time of digestion (Two-way ANOVA, F = 2.50, P>0.05), although a slight non-significant increase was observed from 6 to 12 h after ingestion of wheat and rice diets (Fig. 3c), in correspondence with the decrease found in soluble protein during this period (Fig. 2a). Free amino acid levels differed among experimental diets (Two-way ANOVA, F = 26.10, P≤0.001) being smaller in maize fed lobsters respect to the other group (Fig. 3c), while there was no interaction between diet and time (Two-way ANOVA, F = 2.82, P>0.05). On the other hand, free amino acid concentration in hemolymph did not differ significantly through time (Two-way ANOVA, F = 2.33, P>0.05) although this parameter tended to raise slowly 12 h after feeding for all diets (Fig. 3d). Concentration of amino acid in hemolymph differed among diets (Two-way ANOVA, F = 4.43, P≤0.05) (Fig. 3d), while there was no interaction between diet and time (Two-way ANOVA, F = 0.77, P>0.05).

### Time-course of triglyceride in gastric juice and hemolymph after feeding

Triglyceride concentration in the gastric juice did not vary significantly through time of digestion (Two-way ANOVA, F = 1.55, P>0.05) but differed among experimental diets (Two-way ANOVA, F = 9.32, P≤0.001), and the interaction between both factors was also significant (Two-way ANOVA, F = 2.86, P≤0.05) (Fig. 3e). Concentration of triglyceride in the gastric chamber of lobsters was lowest with the maize diet (Fig. 3e). There were differences among fed and unfed lobsters (Two-way ANOVA, F = 8.71, P≤0.01) in the triglyceride levels in hemolymph after feeding, although not among formulated feed treatments (Fig. 3f). Yet, non-significant higher values for triglyceride were observed 12 h after ingestion in the wheat diet (Fig. 3f). For all diets, triglyceride concentration in hemolymph varied significantly through time (Two-way ANOVA, F = 15.68, P≤0.001) (Fig. 3f), while the interaction between diet and time was not significant (Two-way ANOVA, F = 1.75, P>0.05). Triglyceride concentration in the hemolymph rose abruptly 12 h after feeding in rice- and maize-fed lobster, while it rose earlier in wheat-fed lobsters (Fig. 3f).

### Lactate in hemolymph after feeding

**Figure 4:**
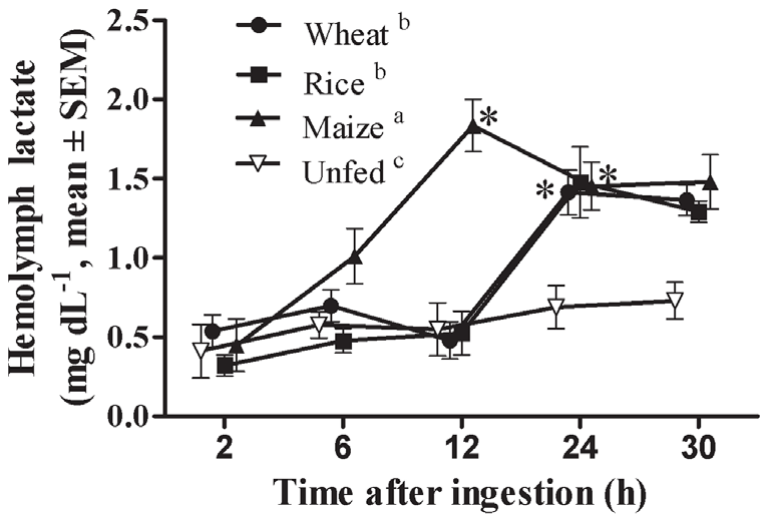
Changes in concentration of lactate in hemolymph of *Panulirus argus* after feeding diets with different carbohydrate sources. Diets were named according to the carbohydrate source they contained (wheat flour, rice starch, maize starch). Each value is the mean ± SEM (N = 5 lobsters per diet). For each dietary treatment, the time at which the first statistically difference was found with respect to 2 h is marked by an asterisk. Differences among diets throughout the 30-h experiments are marked by different superscript letters in the legend (Two-way ANOVA, Tukey test, P≤0.05).

After ingestion, differences were found in lactate concentration in hemolymph among diets (F = 12.89, P≤0.001) and throughout time (F = 32.05, P≤0.001) (Fig. 4). The interaction between both factors was also statistically significant (F = 4.42, P≤0.001). While in rice- and wheat-fed lobsters the concentration of lactate increased in hemolymph 12 h after ingestion, in lobsters that ingested the maize diet it started to increase earlier, attaining 12 h after ingestion the highest values recorded for all diets (Fig. 4).

### Metabolites and metabolic enzymes in tissues of lobsters after feeding different diets

The concentrations of glucose (F = 1.21, p>0.05), glycogen (F = 3.13, p>0.05), lactate (F = 1.52, p>0.05), and amino acid (F = 2.17, p>0.05) in the muscle of lobsters did not vary among dietary treatments 24 h after feeding, and differences were only found in triglyceride contents (F = 7.11, p≤0.05), with smaller values for the wheat diet (Tukey's test, P>0.05) (Table 4). At this time, differences were also found in the concentrations of glucose (F = 15.12, p≤0.05), lactate (F = 15.19, p≤0.05), amino acid (F = 5.64, p≤0.05), and triglycerides (F = 5.18, p≤0.05) in the hemolymph (Table 4). However, most differences in hemolymph were observed between fresh fish and formulated feed fed lobsters, without significant differences (Tuckey's test, P>0.05) among formulated feeds, except for triglycerides (Table 4). In the digestive gland, differences were found among dietary treatments in contents of glucose (F = 17.50, p≤0.05), glycogen (F = 8.66, p≤0.05), lactate (F = 30.77, p≤0.001), amino acid (F = 6.27, p≤0.05), and triglyceride (F = 6.94, p≤0.05) (Table 4). However, the only significant differences among groups were a smaller glycogen content in the gland of maize-fed lobsters (Tukey's test, P>0.05), and a low triglyceride content in wheat fed animals (Tukey's test, P>0.05) (Table 4).

**Table 4:** Metabolite levels in digestive gland, muscle and hemolymph of the spiny lobster *Panulirus argus* 24 h after feeding diets with different carbohydrate sources, or fed with fresh fish under the same experimental conditions.

Metabolites	Digestive gland			
	Fresh fish	Wheat	Rice	Maize
Glucose (mg g^−1^)	1.13±0.053^a^	0.41±0.076^b^	0.49±0.097^b^	0.24±0.106^b^
Glycogen (mg g^−1^)	0.04±0.004^a^	0.03±0.007^a^	0.04±0.005^a^	0.01±0.001^b^
Lactate (mg g^−1^)	0.52±0.048^a^	0.16±0.019^b^	0.17±0.028^ b^	0.13±0.040^b^
Amino acid (mg g^−1^)	38.16±3.342^ a^	20.23±1.212^b^	37.56±6.314^ ab^	16.68±2.813^ b^
Triglyceride (mg g^−1^)	91.71±18.870^a^	16.26±7.703^b^	34.44±17.580^ab^	106.26±19.210^a^
	**Muscle**			
Glucose (mg g^−1^)	4.27±0.580	3.61±0.455	4.82±0.303	4.21±0.511
Glycogen (mg g^−1^)	0.23±0.017	0.14±0.033	0.13±0.012	0.19±0.032
Lactate (mg g^−1^)	5.90±1.075	3.49±0.833	4.00±0.369	3.84±0.745
Amino acid (mg g^−1^)	37.74±3.864	26.19±3.683	28.70±2.294	27.66±5.242
Triglyceride (mg g^−1^)	1.30±0.194^a^	0.55±0.133^b^	0.69±0.114^ab^	1.41±0.130^a^
	**Hemolymph**			
Glucose (mmol L^−1^)	0.53±0.043^a^	1.12±0.195^b^	1.401±0.092^b^	1.04±0.110^b^
Lactate (mg dL^−1^)	2.89±0.237^a^	1.41±0.140^b^	1.48±0.222^b^	1.45±0.151^b^
Amino acid (mmol dL^−1^)	3.67±0.715^a^	1.15±0.311^b^	2.01±0.376^ab^	1.44±0.383^b^
Triglyceride (mg dL^−1^)	34.42±14.110^a^	7.958±0.946^b^	13.27±2.150^a^	14.13±1.325^a^

Diets were named according to the carbohydrate source they contained (wheat flour, rice starch, maize starch). All data are expressed on a dry matter basis. Each value is the mean ± SEM (N = 5 lobsters per diet). Different letters in the same row indicate significant differences among groups (one-way ANOVA, Tukey test, P≤0.05).

Enzymes that differed in activity among dietary treatments 24 h after feeding were (Table 5): G3PDH (F = 6.367, p≤0.05), FBP (F = 3.964, p≤0.05), GPasa (F = 3.382, p≤0.05), and AST (F = 9.922, p≤0.05) in muscle, and PK (F = 5.04, p≤0.05), LDH (F = 4.482, p≤0.05), FBP (F = 4.635, p≤0.05), G6PDH (F = 106.8, p≤0.0001), AST (F = 6.355, p≤0.05), and HOAD (F = 5.463, p≤0.05) in the digestive gland. No significant differences were found in the activity of the following enzymes: HK (F =  0.271, p>0.05), LDH (F = 0.489, p>0.05), G6PDH (F = 0.583, p>0.05), ALT (F = 0.738, p>0.05), GDH (F = 0.728, p>0.05), HOAD (F = 2.565, p>0.05) in muscle, and G3PDH (F = 0.633, p>0.05), ALT (F = 1.13, p>0.05) and GDH (F = 1.248, p>0.05) in the digestive gland.

**Table 5:** Activities of key enzymes of intermediary metabolism in digestive gland and muscle of the spiny lobster *Panulirus argus* 24 h after feeding diets with different carbohydrate sources, or fed with fresh fish under the same experimental conditions.

Route/Enzyme	Digestive gland				Muscle			
	Fresh fish	Wheat	Rice	Maize	Fresh fish	Wheat	Rice	Maize
Glycolysis								
HK	nd	nd	nd	nd	0.45±0.177	0.74±0.336	0.58±0.217	0.51±0.218
G3PDH	14.79±2.124	18.46±5.143	10.76±2.475	17.63±6.625	5.99±0.927^a^	6.97±0.894^a^	2.50±0.479^b^	3.05±0.415^b^
PK	32.25±3.301^ab^	35.29±4.078^a^	22.00±4.314^ab^	16.91±2.590^b^	nd	nd	nd	nd
Glycogenesis								
LDH	940±106^a^	992±141^a^	730±161^ab^	326±103^b^	1.99±0.459	1.42 ± 0.194	1.85±0.319	2.12±0.354
FBPase	4.00±0.741^ab^	5.05±0.360^ab^	2.53±0.790^a^	6.40±0.765^b^	9.58±1.415^ab^	13.38±2.604^a^	10.47±0.948^ab^	4.46±1.200^b^
Glycogenolysis								
GPase	nd	nd	nd	nd	165.6±21.72^a^	131.0±16.46^ab^	87.90±6.868^b^	122.2±25.07^ab^
Pentose shunt								
G6PDH	2.04±0.414^a^	39.84±4.080^b^	2.61±1.100^a^	4.57±1.351^a^	1.33±0.333	1.68±0.418	1.26±0.337	0.96±0.075
Amino acid								
AST	368.6±63.68^a^	293.4±43.52^a^	709.95±79.67^b^	547.05±146.700^ab^	7.24±1.050^a^	12.89±1.936^a^	32.46±4.322^b^	14.36±6.379^a^
ALT	11.4±1.562	15.80±1.460	14.7±1.319	14.3±0.775	6.35±0.767	7.63±0.766	7.68±0.865	6.81±0.553
GDH	360.82±57.30	396.5±46.45	263.8±49.34	278.0±74.54	17.08±2.709	16.47±3.157	12.68±1.112	18.61±4.665
Fatty acid								
HOAD	181.9±37.09^ab^	296.4±42.54^b^	133.4±30.23^a^	120.6±23.79^a^	5.53±0.890	6.87±0.938	5.03±0.606	3.21±1.002

Diets were named according to the carbohydrate source they contained (wheat flour, rice starch, maize starch). All enzyme activities are expressed as U mg protein^−1^. nd: not detected. Each value is the mean ± SEM (N = 5 lobsters per diet). For each tissue, different letters in the same row indicate significant differences among groups (one-way ANOVA, Tukey test, P≤0.05).

### Discriminant analysis of metabolic enzymes and metabolites

**Figure 5:**
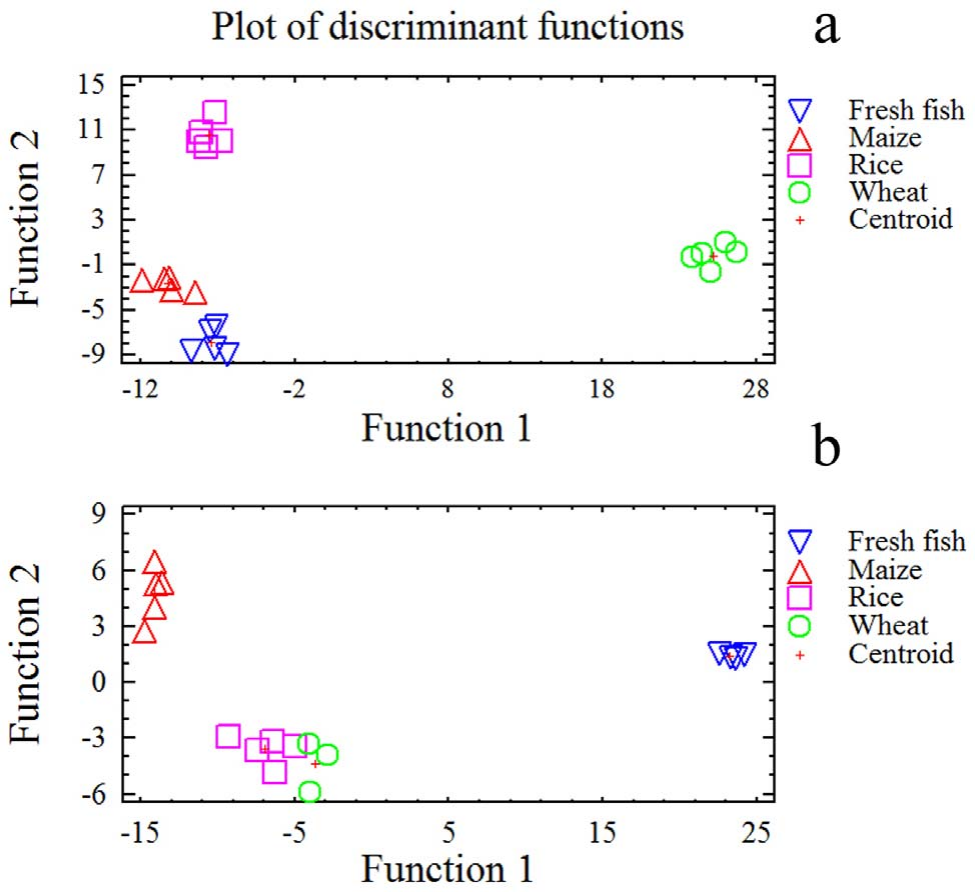
Plots of the first two axes from the forward stepwise discriminant function analysis of metabolic enzymes (a) and metabolites (b) in lobster, *Panulirus argus*, ingesting diets with different carbohydrate sources or left unfed under the same experimental conditions. Several metabolic enzymes and metabolites from the digestive gland, the muscle and the hemolymph were included in the analysis (see text for details). Diets were named according to the carbohydrate source they contained (wheat flour, rice starch, maize starch).

Three significant discriminant functions could be developed from the activity of metabolic enzymes included in the analysis. The first two functions collectively accounted for 96% of the total variance (Table 6). Specific activities of G6PDH in the digestive gland, and FBPase and GPase in muscle were the variables with the highest relevance in the first discriminant function (Table 7), in which wheat fed lobsters showed central values as opposed to those of the lobsters ingesting the other formulated diets or fresh fish (Fig. 5a, axis *x*). On the other hand, activities of PK, FBPase and HOAD in the digestive gland, and G3PDH, FBPase, AST and GPase in muscle had the highest weight in the second discriminant function (Table 7), which discriminated among all dietary treatments but wheat (Fig. 5a, axis *y*). No function built from metabolic enzyme activities could strongly discriminate between fresh fish or maize fed lobsters (Fig. 5a).

**Table 6:** Summary of the canonical discrimination analysis for discriminant functions used to identify the experimental diet ingested by lobster, *Panulirus argus*.

Variable/Function	Eigen value	Percentage of variance	Canonical correlation	Wilks Lambda	Chi-Square	d.f.	P
**Enzymes**							
1	266.30	79.09	0.99	0.04×10^−4^	148.26	30	<0.001
2	56.22	16.70	0.99	0.001	81.20	18	<0.001
3	14.18	4.21	0.97	0.065	32.64	8	<0.001
**Metabolites**							
1	284.49	93.09	0.99	0.04×10^−3^	100.67	30	<0.001
2	17.72	5.80	0.97	0.012	44.13	18	<0.001
3	3.41	1.11	0.88	0.227	14.83	8	>0.05

**Table 7:** Standardised function coefficients for each of the significant discriminant functions used to identify the experimental diet ingested by lobster, *Panulirus argus*.

Variable	Function		
	1	2	3
**Enzyme (tissue)**			
PK (DG)	−0.024	**−1.347**	**1.326**
LDH (DG)	−0.146	−0.544	0.364
FBP (DG)	−0.422	**−1.274**	**−0.739**
G6PDH (DG)	**1.369**	0.417	−0.393
AST (DG)	−0.482	−0.571	0.082
HOAD (DG)	0.497	**−2.289**	**1.244**
G3PDH (M)	−0.012	**1.344**	−0.117
FBPase (M)	**1.195**	**1.043**	0.262
AST (M)	−0.487	**1.903**	−0.351
GPase (M)	**−1.829**	**−2.797**	−0.549
**Metabolites (tissue)**			
Glycogen (DG)	**1.770**	−0.470	-
Glucose (DG)	**2.560**	**−1.306**	-
Lactate (DG)	**2.702**	−0.247	-
Triglyceride (DG)	**−1.742**	**1.570**	-
Amino acid (DG)	−0.130	0.083	-
Triglyceride (M)	0.659	−0.022	-
Glucose (Hph)	**−1.161**	−0.622	-
Lactate (Hph)	0.812	0.207	-
Triglyceride (Hph)	−0.501	**0.793**	-
Amino acid (Hph)	**−2.123**	0.564	-

Coefficients with more weight inside the discriminant function are in bold. DG, digestive gland; M, muscle; Hph, hemolymph; PK, pyruvate kinase; LDH, L-lactate dehydrogenase; FBPase, fructose 1,6-biphosphatase; G6PDH, glucose-6-phosphate dehydrogenase; AST, aspartate transaminase; HOAD, 3-hydroxyacyl-CoA dehydrogenase; G3PDH, glycerol-3-phosphate dehydrogenase; GPase, glycogen phosphorylase.

Only two statistically significant functions were able to predict the diet ingested by lobsters from metabolite data, but the single first function explained most of the variability (93%) (Table 6). The variables that best identified the diet ingested by lobsters were glycogen, glucose, lactate, and triglycerides in the digestive gland, and glucose and amino acid in hemolymph (Table 7). Wheat- and rice-fed lobsters were poorly discriminated by both functions (Fig. 5b).

## Discussion

### Carbohydrate digestibility in the spiny lobster

Methods to assess the *in vitro* digestibility of feedstuffs were developed as alternatives to expensive and time-consuming *in vivo* methods [67]. They are particularly suited when there is no previous information for a particular species, such as *P. argus*, as they allowed for the evaluation of relatively high amounts of feedstuffs.

Most starches contain 72–82% of amylopectin and 18–33% of amylose, but proportions vary according to the botanical origin [68]. In general, starch susceptibility to hydrolysis depends on the amylose content [9], [69]. High amylose content decreases starch digestibility due to a denser packing of the helicoidal structure [70] and the formation of amylose-lipid complex [7]–[9]. In addition, starch hydrolysis is also affected by the granule size (i.e., more available surface area for enzyme action in small granules) [10], [71], [72], and type of crystalline polymorphic form [73]. Rice, wheat, and maize starches display the A-type with amylopectins of relatively short branch chains, while potato starch has amylopectins of long branched chains (B-type). In general, A-type starches are more susceptible to α-amylase, especially those of rice and wheat ([73], and reference therein).

Rice starch is known to be highly digestible due to its small granule size [74], [75] and A-type [73]. Accordingly, native rice starch presented the highest *in vitro* hydrolysis rate in this study (Fig. 1). For this reason, it is not surprising that the rice flour was also hydrolyzed at a high rate, even higher than other purified starches tested (Fig. 1). Rice flour was the only carbohydrate source tested that produced more free glucose than the expected from the correlation between glucose and maltose released, indicating that products of digestion of rice are better substrates for glucosidase enzymes than the products of hydrolysis of the other carbohydrates studied. Following rice starch, major hydrolysis rates were obtained for gelatinised starches of potato and maize (Fig. 1). During gelatinisation (i.e. heating of starch in presence of water), temperature increases the molecular mobility of pre-hydrated and swelling amorphous regions of starch granule and unravels the double helices of amylose, converting the crystalline material into amorphous/gel material [76] thus increasing digestibility [77]. As in other crustaceans [1], [18], [78], gelatinisation increased starch digestibility in spiny lobsters such as *J. edwardsii* [2], [3] and *P. argus* (this study).

Nutritional studies in fish [79] and crustaceans (*Litopenaeus vannamei* [1], *Jasus edwardsii* [2], *Homarus gammarus* [18]) provided evidence of the high digestibility of native wheat starch. It is known that high digestibility of native wheat starch is due to the high amylopectin content (∼80%) [30], [80] of its A-type granules [73]. Accordingly, we observed high *in vitro* digestibility for wheat flour (Fig. 1, Table 3). Both the rice and the wheat diets presented high *in vivo* digestibilities without statistical differences (Table 3). On the other hand, maize starch was less digestible *in vitro* and *in vivo* than all other starches studied (Fig. 1, Table 3). Maize starch was not well digested in other crustaceans [81]–[83], including the spiny lobster *J. edwardsii* [2], [3]. Starch from maize has relatively small granules, but a high content of amylose and a polyhedral form, which are two factors that affect hydrolysis negatively [68].

Carbohydrates such as agar, agarose, alginate, and carboxymethyl cellulose (CMC) are often used as binders in formulated feeds [84]. All these carbohydrate sources were deficiently digested by *P. argus* (Fig. 1). Poor *in vitro* digestibilities were also reported for agar and alginate in the spiny lobster *J. edwardsii* [2], and they produced poor growth results when included in diets for this species [4]. *In vitro* hydrolysis rate of CMC, a derivative of cellulose, was poor in *P. argus*, in agreement to reports in other crustaceans [17], [18], [85], [86]. However, CMC was notably more digestible in *J. edwardsii*, with hydrolysis rates comparable to those of the gelatinised starches and glycogen, and even higher than maize and potato starches [2], suggesting difference between this lobster species and *P. argus* on the presence or activity of cellulase (e.g. endo-β-1,4-glucanase and β-1,4-glucosidase) and hemicellulase (e.g. laminarinase) enzymes. Glucosidase and laminarinase activities were reported for *J. edwardsii* [20], but they have not been studied to date in *P. argus.*

### Time-course of carbohydrate digestion in the spiny lobster

Soluble proteins in the gastric juice of *P. argus* increased during the first 6 h of digestion (Fig. 2a), probably due to both the dissolution of dietary proteins, and the secretion of digestive enzymes that occurs within the first 4 h after ingestion [12], [51]. Six hours after feeding, the concentration of soluble proteins in the gastric juice of lobsters fed the maize diet was significantly lower than in lobsters fed the other diets (Fig. 2a) in correspondence with the low *in vivo* protein digestibility obtained for this diet (Table 3). A second peak of soluble protein in the gastric juice was observed 24 h after feeding in all experimental groups (Fig. 2a). A highly similar pattern of soluble protein in the gastric juice after ingestion of formulated diets was observed in the spiny lobster *J. edwardsii*, and this second peak probably results from the secretion of digestive enzymes in preparation for the next feeding [12].

Although no significant differences were found in gastric juice amylase activity after consuming the experimental diets (Fig. 2b), some secretion of amylase enzymes should occur during the first 6 h for the activity to remain at the same level despite the dilution effect caused by sea water intake during and after ingestion [12], [51]. Significant secretion of amylase enzymes was found in *J. edwardsii*, but in both *J. edwardsii* [12] and *P. argus* [51], higher secretion occurred for other digestive enzymes (e.g., trypsin). High amylase activity in the gastric juice of fasted *P. argus* [21] may suffice to face the small content or relatively high digestibility of carbohydrate in its natural diet (i.e., glycogen), although some adaptation of amylase enzymes to dietary carbohydrates was reported in spiny lobsters [5], [6].

The three tested diets induced similar patterns and levels of free glucose in the gastric juice of lobsters (Fig. 3a). In general, most intense hydrolysis of carbohydrates began 2 h after ingestion to peak 6 h after meal. Gastric juice glucose levels decreased after 6 h in lobsters ingesting the rice and maize diets, while in lobsters fed on the wheat diet, glucose concentration started to decrease later (i.e., 12 h after meal) (Fig. 3a). Although measured glucose in the gastric juice may be impacted by several factors (e.g. sea water ingestion, secretion of gastric juice, sampling artifacts) these results indicate that carbohydrates in formulated diets are highly hydrolyzed within the first 6 h after ingestion. In accordance, the increase of free glucose in hemolymph started at 6 h post-ingestion with maximal glycemic response from 12 to 24 h after feeding (Fig. 3b). The glycemic response of *P. argus* to ingestion of formulated diets tested was high and remained for 30 h, as reported in other lobster species [2], [4], [56]. However, some difference could be noted in glycemic response among diets. Glucose increased at the similar rate in hemolymph of lobsters that ingested the rice and wheat diets, although the peak was higher for the rice diet (Fig. 3b) in agreement with its high rate of *in vitro* hydrolysis (Fig. 1). Rice and maize diets produced maximal glycemic responses within the first 12 h after feeding (Fig. 3b). On the other hand, the wheat diet induced a more gradual increase in hemolymph glucose, with a low glycemic response during the first 12 h after meal, and a rapid enrichment in hemolymph glucose from 12 to 24 h (Fig. 3b). This result matches with a slower clearance of glucose from the gastric fluid in wheat-fed lobsters (Fig. 3a).

The main difference in the time-course of carbohydrate digestion between tropical *P. argus* and temperate *J. edwardsii* is a more rapid glycemic response in the tropical species, maybe related with a higher metabolic rate [87]. Maize starch in formulated diet produced maximal glycemic response 24 h after ingestion in *J. edwardsii* [2] and after 12 h in *P. argus* (this work).

### Dietary carbohydrate and digestion of other nutrients in the spiny lobster

Dietary protein hydrolysis proceeded more slowly than carbohydrates as judged for by the non-significant increase of free amino acid in the gastric juice of *P. argus*, although more intense hydrolysis was apparent 6–12 h after ingestion (Fig. 3c). This is in accordance with previous observations of secretion of proteases (e.g. trypsin) from the digestive gland into the gastric chamber 4 h after ingestion in this species [51] and in *J. edwardsii* [12]. The low concentration of free amino acid observed in the gastric juice of maize-fed lobsters (Fig. 3c) also corresponds with the low *in vivo* protein digestibility of this diet (Table 3). The mechanism by which dietary carbohydrate exerts this effect on protein digestibility in lobsters remains unknown. Amino acids tended to increase at 12 h post-ingestion (Fig. 3d) in correspondence with the clearance tend observed in the gastric juice. However, there were no differences in amino acid concentration in hemolymph through time, and this result may be related with deficient digestion of formulated diet proteins in spiny lobsters [50], or with intense amino acid utilization [31], [32]. The highest protein digestibility in *P. argus* was 83% for the wheat diet (Table 3), as reported before for other fish meal-based diets for spiny lobsters (83% [39], 84% [88], 82–88% [3]), although higher values (∼97%) were reported in *J. edwardsii* for a diet containing more water soluble ingredients such as sodium caseinate and protein hydrolysates [3].

Results from several growout studies [40], [45], [89] indicated that lipid digestion and/or utilization in spiny lobsters fed formulated diets may be compromised. Lipid accumulation in the digestive gland of *J. edwardsii* was poor even when ingesting high lipid content diets [45]. The results from this study suggest that triglyceride solubilization/emulsification in the gastric chamber of lobsters may be not intense, although a non-significant tend to increase was noted with maximal values 12 h after ingestion (Fig. 3e). In addition, the analysis of triglyceride content in hemolymph indicated differences among diets on lipid digestion and/or utilization. Lipid in the wheat diet seems to be hydrolysed, and fatty acids re-esterified, earlier as its concentration in hemolymph rose before than with the other diets (Fig. 3f). Conversely, lipid from the maize and rice diets took up to 12 h for appearing in hemolymph as triglycerides, and then rose abruptly (Fig. 3f) suggesting a delay in digestion/re-esterification and a poor metabolic use. This was observed before in spiny lobsters, whereby in *Panulirus interruptus* fed ^14^C-labeled triglyceride, radioactivity was not seen in the hemolymph until 12 hr after feeding [90]. However, it is known that most lipids in the hemolymph of spiny lobsters are associated with high density lipoproteins with only 3% triglyceride, in contrast with 88% phospholipid, mostly phosphatidyl choline (*P. interruptus* [90]), thus the effect of feeding on hemolymph phospholipid deserves further studies. Still, high post-prandial hypertriglyceridemia observed in all treatments contrasts with low lipid accumulation in the digestive gland of lobster (Table 4). In insects, diet-derived triglyceride, phospholipid, diacylglycerol, and free fatty acids are rapidly cleared from hemolymph to be used for triglyceride synthesis in the fat body [91]. As prolonged hypertriglyceridemia in lobster indicates a decreased ability of tissues to remove plasma triacylglycerol, the activity of tissue lipoprotein lipase is worthy to be studied further. Also, phospholipase activity should be included in future assessments since it could be required to allow access of the lipase to circulating triglyceride. This approach may help to depict the mechanism behind the limited ability of *P. argus* to store lipids from formulated feeds in the digestive gland (this study), and use circulating lipids to spare dietary amino acid from oxidation [32], [87].

Results obtained on the relationship between dietary carbohydrate and lipid utilization in lobster were most intriguing. Diets that produced the highest hyperglycemic responses to feeding produced the higher hypertriglyceridemia as well (Fig. 3b, f). Data obtained in rainbow trout indicated that poor dietary carbohydrate utilization in fish may be related to increased hepatic glucose production (i.e., up-regulation of G6Pase) under conditions of high dietary fat intake [92]. This may be not a plausible explanation for our data, since experimental diet contained just 10% lipid and we found no evidence of increased gluconeogenesis after feeding (Table 5). A more plausible explanation is that high carbohydrate digestion and absorption interferes with lipid utilization as occurs in humans: when the content of dietary carbohydrate is elevated above the level typically consumed, blood concentrations of triglycerides rise even with low-fat diets. This phenomenon is known as carbohydrate-induced hypertriglyceridemia, and is still poorly understood but assumed to be due to increased lipogenesis in liver and limited clearance from blood of both triglycerides [93] and glucose [94]. Postprandial hypertriglyceridemia in humans is also a rare hereditable disorder. As carbohydrate-induced hypertriglyceridemia, it is produced by an amplified production and constrained clearance of triglyceride from plasma, related with an abnormally high expression and activity of hepatic fatty acid synthase, and a decrease in lipase activity in blood and other tissues (i.e., hydrolysis of lipoprotein triglycerides) [95]. It would be worthy to test if this phenomenon does not occur in crustaceans for which no problems have been reported on the utilization of dietary lipid (e.g., penaeid shrimp) in order to clarify the causes of poor lipid utilization in spiny lobsters.

### Dietary carbohydrate and metabolism in the spiny lobster

It is known that in crustaceans carbohydrates exhibit a relatively slow incorporation to the citric acid cycle [34], [96]. Some enzymes involved in glycolysis had smaller activity in the muscle (G3PDH) or in the digestive gland (PK) of maize- and rice-fed lobsters, than in specimens under the wheat treatment, which presented low but similar glycolytic enzyme activity than in fresh fish-fed animals (Table 5). Altogether, these results lead us to postulate that lobster tissues are not able to increase the use of circulating glucose as a source of energy 24 h after feeding despite hyperglycemia persisting at this time and onward. However, as PK catalyzes the formation of pyruvate [97] (and pyruvate might be used in lipid and amino acid metabolism as well), PK activity may be indicative of pathways other than carbohydrate metabolism [36], [98]. Yet, our hypothesis is strengthened by the facts that no detectable HK activity was found in the digestive gland of lobsters and that no differences were found in muscle HK activity of lobsters, irrespective of the experimental diet (Table 5). HK phosphorylates glucose to be used by cells, it has been identified as one of the major control sites in glycolysis [99], and is known to be indicative of preferential use of free glucose [35], [65]. HK activity was not detected in muscle of larval or postlarval stages of the spiny lobster *J. edwardsii* [100] but in adults, both HK and PK activities [101] were comparable to those obtained here for intermolt *P. argus*.

An alternative fate of post-prandial circulating glucose other than glycolysis is its storage as glycogen mostly in the digestive gland, but low glycogen content was found in the gland of all lobsters. This result matches with previous observations in this [33] and other spiny lobster species [5], [6], and indicates that lobsters have a limited capacity for glycogen synthesis during the feeding period of intermolt. Given the low amount of glycogen in both the digestive gland and muscle of lobsters (Table 4), it is not surprising that a low glycogenolytic potential (i.e., GPase activity) was found in all treatments (Table 5). Overall, these results indicate that the energy demands in lobster would not be covered by the use of glucose whether from hemolymph or from glycogen mobilization. It is noteworthy, however, that the smallest amount of digestive gland glycogen was observed in maize-fed lobsters. Maize starch was less digestible to lobsters, with respect to rice starch and wheat flour both *in vitro* (Fig. 1) and *in vivo* (Table 3), and this difference in digestibility may impact glycogen synthesis as reported previously in the lobster *J. edwardsii* [5], [6], although a poor capacity to increase gluconeogenesis after feeding is also a plausible explanation. A reduced capacity for gluconeogenesis contributed to depress liver glycogen in Salmon [102]. Actually, LDH activity did not increase after feeding indicating a poor induction of lactate-pyruvate conversion in response to high circulating lactate, most notably in maize-fed lobsters. The analysis of lactate time-course changes in hemolymph revealed that in maize-fed lobsters the capacity to process lactate in the digestive gland would be certainly impaired since lactate accumulated in hemolymph earlier than with the other diets (Fig. 4). It is known that low or no gluconeogenic activity occurs in penaeid shrimp fed diets containing more than 20% carbohydrates [34], as those used in our experiments on lobster. LDH activity in muscle was much lower in *P. argus* muscle with respect to *J. edwardsii* [101].

The source of post-prandial lactate in lobster remains unknown, since there were no differences in the lactate content of the digestive gland, hemolymph, or muscle (Table 4). Also, the metabolic fate of this lactate is poorly understood. In mammals, a significant fraction of lactate is remetabolized to glycogen through gluconeogenesis in the liver and the kidney [103]. In fish, the liver, kidney and muscle have all been identified as potential sites for gluconeogenesis [104], [105]. However, gluconeogenic enzymes activities have been either very low or absent in the different crustaceans studied, suggesting a gluconeogenic pathway other than the Cori cycle [106], [107], [108] or that this cycle proceeds at a very slow rate [109]. Our data are consistent with the fact that other crustaceans cannot excrete or rapidly mobilize this metabolic end product [110], [111]. Although some [^14^C] lactate was incorporated into glycogen in muscle of *Callinectes sapidus*, most of it remained unmetabolized in the hemolymph [109]. It was postulated that in crustaceans, due to the lack of a well-developed central nervous system, there is no selective advantage in the efficient recycling of lactate to glucose [109]. Moreover, lactate is known to cause an increase of hemocyanin oxygen affinity [112] thus high lactate concentration in hemolymph would be even advantageous for the increased oxygen consumption imposed by feeding [32], [87]. While the post-exercise/emersion lactate accumulation in hemolymph is well documented in crustaceans, we show that lactate is produced under normoxic conditions in lobster as a consequence of low glycolytic and gluconeogenic potentials after feeding.

Lobsters fed maize and rice diets seem to increase amino acid catabolism, as judged by the higher activity of AST enzyme both in muscle and digestive gland (Table 5), although we could not find higher activity also for GDH enzyme, which is involved in ammonia formation [113], as expected under this scenario [114]. This is probably the first study providing direct biochemical evidence of an effect of dietary carbohydrate on protein metabolism in spiny lobsters.

HOAD, that catalyzes the third step of beta oxidation of fatty acids, had higher activity in *P. argus* than in *J. edwardsii* [101]. In addition, this activity was higher in the digestive gland of wheat-fed lobsters (Table 5), in correspondence with the lower values observed for triglycerides in digestive gland, hemolymph and muscle (Table 4), which indicates a more intense lipolysis and fatty acids utilization. Also, we found higher values for G6PDH activity in the digestive gland of wheat-fed lobsters (Table 5). Since this enzyme is involved in the pentose shunt, which NADPH is needed for various synthetic pathways, including lipid synthesis [115], it can be postulated that the up-regulation of G6PDH observed in digestive gland of *P. argus* specimens responds to the increased use of lipid in wheat-fed lobsters.

Differences found in metabolism may be attributed to the long-term feeding (i.e., one month) with carbohydrate differing in digestion kinetic. Wheat flour was hydrolyzed within the first 6 h of digestion as the other diets, but free glucose was absorbed slowly (i.e., slow clearance of glucose from the gastric juice) producing a moderate and gradual glycemic response, and a delay in the peak to 24 h post-feeding. It is interesting that the kinetics of carbohydrate digestion and assimilation may affect the protein-sparing effect of lipids, instead of carbohydrate. Present results sustain the observation from O:N ratio studies that the potential for protein-sparing in lobsters relies on dietary lipid [32], and these results also indicate that dietary carbohydrate plays a key role in determining the metabolic fate of other nutrients. Since we used a tropical lobster as the experimental model, it is needed to test if these effects are produced also in lobster species with slower metabolic rates (i.e., temperate species), for which apparently there is little effect of carbohydrate source on growth rate [5].

The multivariate analysis in this study supported that lobsters respond to feeding by changing the level of metabolites and activities of some key metabolic enzymes according to the diet ingested. However, while most of the metabolite variables predictive of carbohydrate source ingestion were related with carbohydrate digestion itself and carbohydrate metabolism, the discriminant analysis revealed that the type of carbohydrate ingested has a profound effect on overall metabolism, being the predictable variables involved in all metabolic routes analyzed in this study, from carbohydrate processing routes to amino acid and fatty acid metabolism (Table 7). Results point out that including wheat flour in lobster diet improved lipid processing in the digestive gland, and the formation of glucose in muscle by both gluconeogenesis and glycogenolysis (Table 7), which would positively impact growth rate.

### Prospects for carbohydrate use in *P. argus* formulated feeds

Most studies on the spiny lobster *P. argus* digestion have been focused on protein digestion [21], [22], [50], [51], [116], while the digestion of other nutrients has received less research attention. The improvement of carbohydrate utilization was suggested as one of the key issues to improve the performance of formulated feeds in spiny lobsters [2], [3]. Although several issues of carbohydrate digestion were studied in other spiny lobster species [2], [3], [5], [6], [12], an integrative analysis of dietary carbohydrate digestion and utilization such as the presented in this work has not been available before for *P. argus*.

In general, starches are well digested by spiny lobsters [2,3, this work] and pretreatment of starches is considered to increase their digestibility [2], [18]. We showed that the gelatinisation of starch does improve digestibility in lobster, but this would not necessarily impact their utilization. In fact, carbohydrates that result in a more gradual digestion and liberation of glucose to the hemolymph (e.g., native wheat flour) may have the major potential for optimizing the metabolism of lobster under growout conditions as suggested before [2]. This was evidenced in this study by the better metabolic utilization of carbohydrates of the wheat diet with respect to the rice diet, which contains rapidly digested rice starch. Maize starch is not recommended as a carbohydrate source in lobster diet due to relatively low digestibility and its negative effect on the digestion and utilization of other nutrients. Maize starch does not promote good growth performance in fish due to its poor digestibility [117], although it is well utilized by the omnivorous freshwater crayfish *Procambarus clarkii* [118]. The inclusion of carbohydrates of low digestibility such as CMC in fish diets improves diet utilization [119], as occurs in lobsters [3]. However, the time course absorption of diet-derived glucose seems more important in lobster than overall digestibility, since low digestible maize starch also produces rapid hyperglycemia, thereby having a negative impact on the metabolic use of other nutrients. In summary, although the metabolism of *P. argus* is strongly supported by amino acids to meet energy requirements [32], [87], reorganization of energy metabolism after feeding depends, among other factors, on the extent and time-course of digestion of dietary carbohydrates.
